# Challenges With Continuous Pulse Oximetry Monitoring and Wireless Clinician Notification Systems After Surgery: Reactive Analysis of a Randomized Controlled Trial

**DOI:** 10.2196/14603

**Published:** 2019-10-28

**Authors:** Prathiba Harsha, James E Paul, Matthew A Chong, Norm Buckley, Antonella Tidy, Anne Clarke, Diane Buckley, Zenon Sirko, Thuva Vanniyasingam, Jake Walsh, Michael McGillion, Lehana Thabane

**Affiliations:** 1 Health Research Methods, Evidence and Impact McMaster University Hamilton, ON Canada; 2 Department of Anesthesia McMaster University Hamilton, ON Canada; 3 Western University London, ON Canada; 4 St. Joseph’s Healthcare Hamilton, ON Canada; 5 Hamilton Health Sciences Hamilton, ON Canada; 6 School of Nursing McMaster University Hamilton, ON Canada

**Keywords:** continuous pulse oximetry, wireless notification, issues, evaluation of issues, clinical adoption framework, remote monitoring, postoperative monitoring, false alarm

## Abstract

**Background:**

Research has shown that introducing electronic Health (eHealth) patient monitoring interventions can improve healthcare efficiency and clinical outcomes. The VIGILANCE (VItal siGns monItoring with continuous puLse oximetry And wireless cliNiCian notification aftEr surgery) study was a randomized controlled trial (n=2049) designed to assess the impact of continuous vital sign monitoring with alerts sent to nursing staff when respiratory resuscitations with naloxone, code blues, and intensive care unit transfers occurred in a cohort of postsurgical patients in a ward setting. This report identifies and evaluates key issues and challenges associated with introducing wireless monitoring systems into complex hospital infrastructure during the VIGILANCE eHealth intervention implementation. Potential solutions and suggestions for future implementation research are presented.

**Objective:**

The goals of this study were to: (1) identify issues related to the deployment of the eHealth intervention system of the VIGILANCE study; and (2) evaluate the influence of these issues on intervention adoption.

**Methods:**

During the VIGILANCE study, issues affecting the implementation of the eHealth intervention were documented on case report forms, alarm event forms, and a nursing user feedback questionnaire. These data were collated by the research and nursing personnel and submitted to the research coordinator. In this evaluation report, the clinical adoption framework was used as a guide to organize the identified issues and evaluate their impact.

**Results:**

Using the clinical adoption framework, we identified issues within the framework dimensions of people, organization, and implementation at the meso level, as well as standards and funding issues at the macro level. Key issues included: nursing workflow changes with blank alarm forms (24/1030, 2.33%) and missing alarm forms (236/1030, 22.91%), patient withdrawal (110/1030, 10.68%), wireless network connectivity, false alarms (318/1030, 30.87%), monitor malfunction (36/1030, 3.49%), probe issues (16/1030, 1.55%), and wireless network standards. At the micro level, these issues affected the quality of the service in terms of support provided, the quality of the information yielded by the monitors, and the functionality, reliability, and performance of the monitoring system. As a result, these issues impacted access through the decreased ability of nurses to make complete use of the monitors, impacted care quality of the trial intervention through decreased effectiveness, and impacted productivity through interference in the coordination of care, thus decreasing clinical adoption of the monitoring system.

**Conclusions:**

Patient monitoring with eHealth technology in surgical wards has the potential to improve patient outcomes. However, proper planning that includes engagement of front-line nurses, installation of appropriate wireless network infrastructure, and use of comfortable cableless devices is required to maximize the potential of eHealth monitoring.

**Trial Registration:**

ClinicalTrials.gov NCT02907255; https://clinicaltrials.gov/ct2/show/NCT02907255

## Introduction

### Background

Although the adoption of technology in the hospital environment is slow compared to other fields, there has been a recent increase in digital health solutions proposed for health care issues as technologies improve [1]. With increased workload demands on health care providers, hospitals have turned to technological solutions to improve efficiency and safety of patient care [2]. Patient assessment in a typical postsurgical ward happens only once every four to six hours or, at times, just once during day shifts and irregularly at night [3-5]. This infrequent monitoring, combined with the need for opioids and sedatives and the risk of respiratory depression, may predispose patients postoperatively to more frequent cardiorespiratory arrests (ie, code blues), intensive care unit (ICU) transfers, and the need for resuscitation [6-8]. Early detection is the key to preventing complications [9]. Pulse oximetry, capnography, and wireless remote automated monitoring with clinician notification systems are some of the methods that are being used to support safe patient care in the face of declining clinical staff complements [9-11].

The VItal siGns monItoring with continuous puLse oximetry And wireless cliNiCian notification aftEr surgery (VIGILANCE) study examined the impact of continuous pulse oximetry (CPOX) on the incidence of postoperative respiratory complications [8]. VIGILANCE was an unblinded randomized controlled trial (RCT), targeting noncardiac postsurgical patients (n=2049) at the Juravinski Hospital in Hamilton, Canada. All trial patients with an anticipated length of stay of at least 24 hours and scheduled to stay in one of two surgical wards (E4 and F4) were randomized to either the standard (n=1019) or the intervention arms (n=1030). The standard arm participants received routine monitoring, including assessments every four hours by nurses. The intervention arm patients received continuous monitoring of blood oxygen saturation (SpO_2_) and pulse rate (PR) using a wireless respiratory monitoring system, the Nellcor Oxinet III system (Covidien, Dublin, Ireland), in addition to standard monitoring [8,12]. Both E4 and F4 were mixed surgical wards with postsurgical patients admitted after plastic surgery, mastectomies, general surgery, urology, gynecology, orthopedic, and oncology surgeries*.* Both the wards have 24 beds and have approximately 1100 elective and emergent admissions for surgery per year. On both wards the nurse-to-patient ratio is 1:4.

### Need for Evaluation of Issues and Objectives

The VIGILANCE study represented a timely opportunity for the anesthesia service at our institution to work toward reducing the rate of postoperative respiratory complications [8]. At the time, clinical trials research on electronic Health (eHealth) patient monitoring was a burgeoning field, with little prior experience to draw upon, and the challenges associated with introducing digital health systems into the complex hospital infrastructure were not well studied or appreciated [2,13]. Based on our experience, we found that multiple factors interfered with the implementation and conduct of the VIGILANCE study. The purpose of this report was to engage in a reactive analysis by reflecting on the challenges faced by the VIGILANCE research team during the project implementation, followed by identification and evaluation of issues to facilitate future improvements [14]. Through examination of the issues and challenges we faced, our overall aim was to help foster understanding of the difficulties related to eHealth implementation and prevent future implementation challenges [2]. In so doing, our specific objectives were to: (1) identify issues related to deployment of the eHealth intervention system of the VIGILANCE study; and (2) evaluate the influence of these issues on intervention adoption.

## Methods

### Vital Signs Monitoring with Continuous Pulse Oximetry and Wireless Clinician Notification After Surgery: Setting and Structure of the Intervention and Network

**Figure 1:**
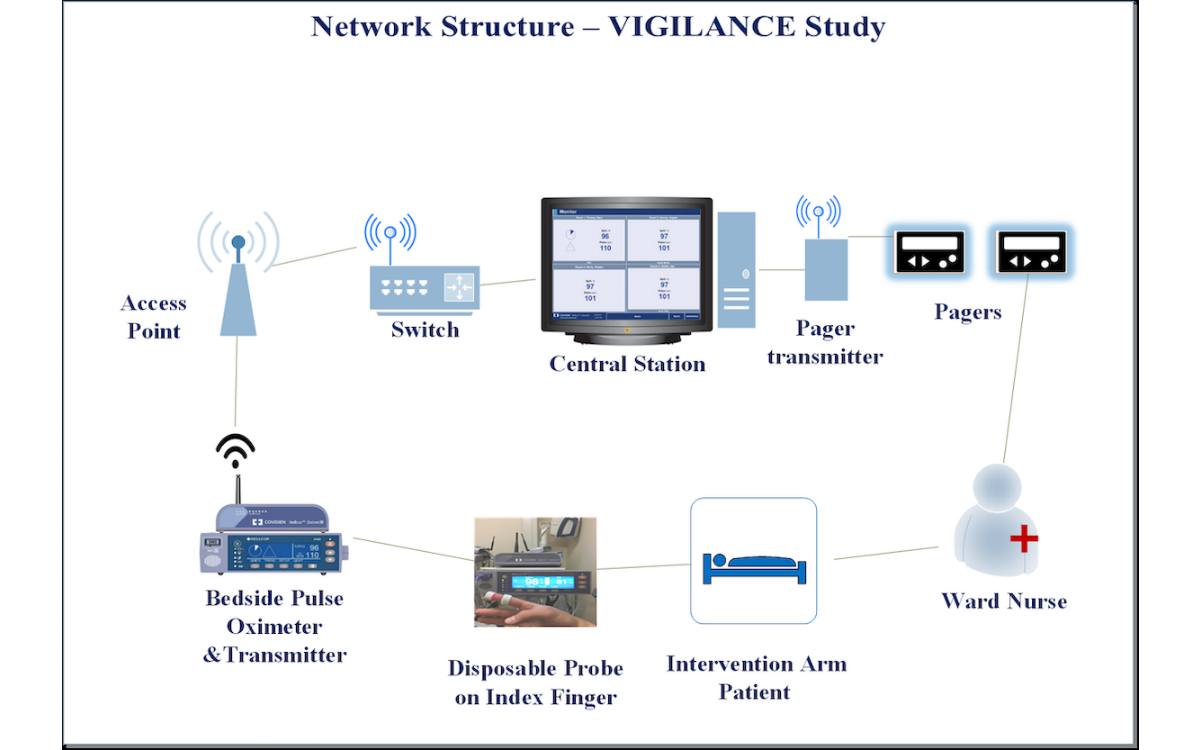
Network Structure of the monitoring system of the VIGILANCE Study. VIGILANCE: VItal siGns monItoring with continuous puLse oximetry And wireless cliNiCian notification aftEr surgery.

The monitoring system within the intervention arm allowed for bedside monitoring and wireless pager notification of clinical staff when the alarm threshold was exceeded. Alarms were set at a threshold of SpO_2_<90% and PR of ≤50 beats per minute or ≥130 beats per minute, to set a balance between safety and false alarms [8]. The network structure of the monitoring system was comprised of probe, pulse oximeter unit, transmitter, an optional monitor stand, access points, wireless network, switch, central station, pager transmitter, and pagers (Figure 1) [12,15].

**Figure 2:**
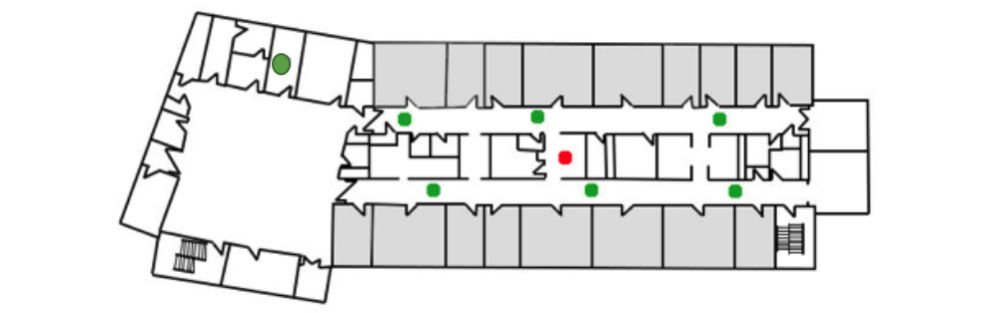
Access points in E4. Green dots: access points in E4 ward; Red dot: central station; Grey rooms: indicate patient rooms; Unfilled/white rooms: other rooms or spaces.

**Figure 3:**
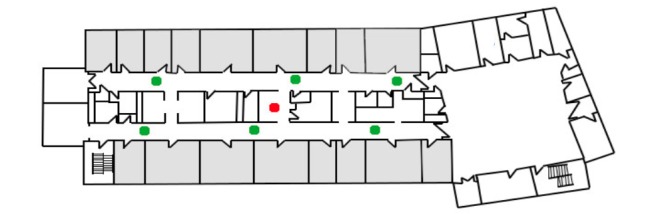
Access points in F4. Green dots: access points in F4 ward; Red dot: central station; Grey rooms: indicate patient rooms; Unfilled/white rooms: other rooms or spaces.

The oximetry probe on the patient’s finger was connected to the bedside CPOX monitor through a cable. The CPOX monitor sent patient data through a wired port to the transmitter. The transmitter then converted the data into Ethernet data and wirelessly sent it to the central station via access points. The hospital wireless network structure was made up of the Institute of Electrical and Electronics Engineers (IEEE) standards 802.11a and 802.11g. The IEEE 802.11a standard provided up to 54 megabits per second (Mbps) in a 5 gigahertz (GHz) band, whereas IEEE 802.11g used a 2.4 GHz band [16]. During the installation of the Wireless Local Area Network (WLAN), the Health Information Technology Services (HITS) staff installed the access points after assessing the wireless connectivity, size of the rooms, and structure of the wards [17]. There were seven access points forming a WLAN on the E4 surgical ward (Figure 2) and six access points on the F4 ward (Figure 3).

Information from the access points was wirelessly sent to the hospital’s internet routers or switch, which were connected to the central station on their respective wards. The patient data, including the patient name, SpO_2_ (%), and PR (beats per minute) along with alarm details, were displayed to the healthcare personnel at their respective central stations. The central station served as the application server and had the Oxinet III software that was required to read the information from the CPOX [12].

### Implementation Measures

Testing of the connectivity of the pulse oximeter and the central monitor to the WLAN was done, and the channels were adjusted according to their connectivity after the study was initiated. Prior to study initiation, training and in-service sessions with the continuous monitoring system were held to train the ward nurses. Research nurses visited the ward daily to provide support and to collect the study forms. To reduce the incidence of false notifications due to transient events, notifications were only sent to the nurses after 30 seconds of the event, with a delay of 15 seconds set for both pager notifications and the bedside monitor [8]. Prior to the study starting, the research ethics board application required key personnel of all the involved hospital areas, including the nursing managers of the study wards, to assess the study requirements and comment back to the originator.

### Design and Conceptual Framework

The presentation of findings in this evaluation report has been guided by Lau et al’s Clinical Adoption (CA) framework [18,19]. The CA framework is an extension of the benefits evaluation framework by Canada Health Infoway, and it is designed to lend guidance to understanding factors, influencing eHealth intervention adoption, in healthcare organizations at macro, meso, and micro levels [18-20]. The overall rationale behind this framework is that, for the successful clinical adoption of technology, the various factors in the framework need to be managed efficiently [18,19]. An underlying premise is that the lower the quality of the technology, as defined by decreased functionality, performance, security, content, availability, and responsiveness, there is an associated decrease of usage, user satisfaction, and acceptance by the stakeholders, and thus overall decreased net benefits [19]. Therefore, this framework was used to understand and organize the various challenges faced during the VIGILANCE study.

For this evaluation, selective constructs were used depending on the issues identified and the context of the project [19,21]. The people, organization, and implementation issues at the meso level were identified [19], and the healthcare standards and funding constructs were included at the macro level [19]. At the micro level, system, information, service quality, use, and net benefits in terms of care quality, access, and productivity, were evaluated [19].

### Data Source

During the conduct of the VIGILANCE study, some of the issues that affected the eHealth intervention arm were documented in the case report forms, alarm event form, and nursing user feedback questionnaire. The VIGILANCE study case report forms included items pertaining to patients’ deviation from the assigned intervention, the evidence of the type of monitoring received and the reasons for patient withdrawal of the study intervention. These data were captured through the Research Electronic Data Capture (REDCap) system [22]. An alarm event form was used to capture details of alarms and the nursing response to these. Nurses who were assigned to intervention patients completed these forms when patients had any true or false alarms and documented the associated symptoms, along with measures taken to address the alarms. Once the patient was discharged, these alarm event forms were deposited in the study storage box and collected by the research nurse, before being deidentified, scanned, and saved in the study folder in Dropbox [23]. Forms that were not deposited were scanned, along with the patient chart, into the SOVERA (CGI Inc, Montreal, Quebec) hospital health record storage system. Nursing user feedback surveys were also administered to ward nurses after completion of the VIGILANCE study and will be reported in a separate study. Any other issues related to the VIGILANCE pulse oximeter, wireless network connectivity, or nursing workflow, as experienced by the ward staff and the research personnel, were reported to the study coordinator on an ongoing basis.

### Data Analysis

Data analysis involved identification of issues from the data sources, categorization of these issues under the meso and macro level of the CA framework, and evaluation of the impact of these on the micro level constructs of the CA framework by reflecting on the VIGILANCE study happenings during discussions within the study team [14,19]. The problems identified at the meso level were described under people, organization, and implementation categories, and those identified at the macro level were described under standards and funding categories [19]. The impact of these issues on the micro level factors of the CA framework was described under quality, usage, and net impact categories [19]. The identified issues were quantified and are presented using descriptive statistics generated through REDCap, along with counts and percentages for these issues.

## Results

### Summary

**Figure 4:**
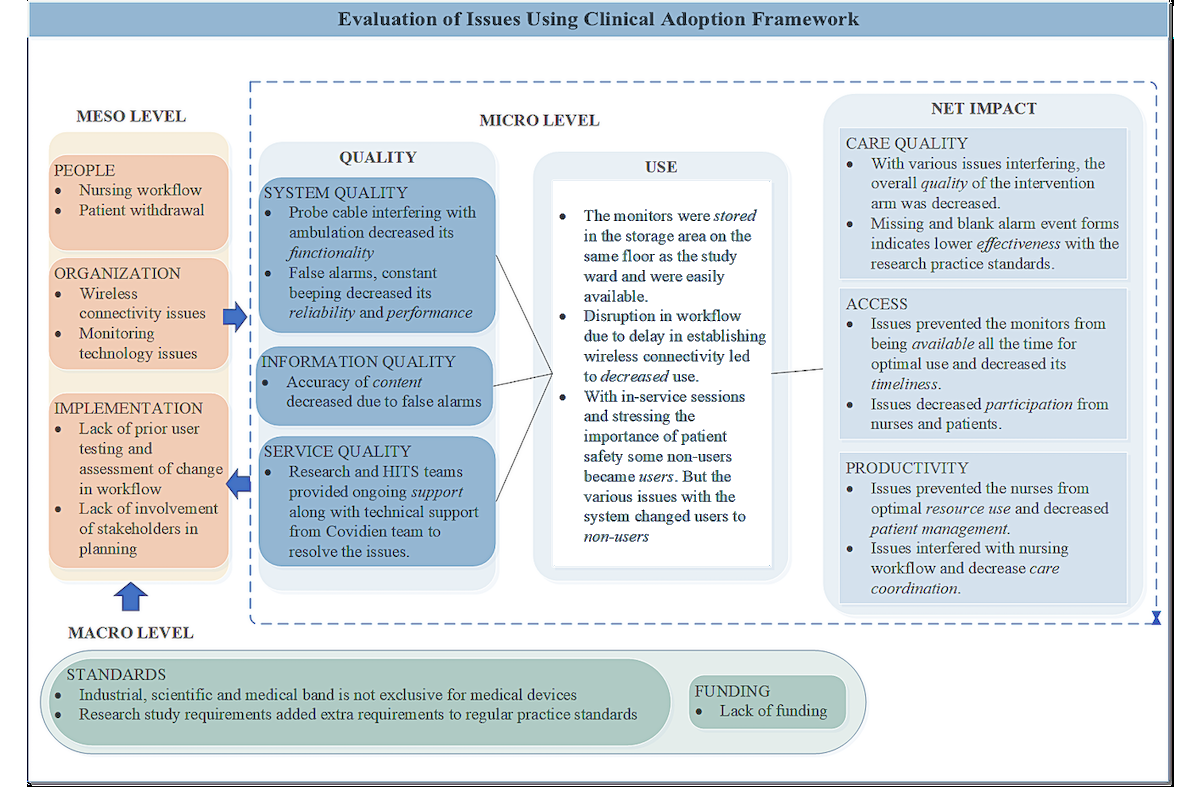
Evaluation of issues using the clinical adoption framework. HITS: health information technology services.

This evaluation report used the CA framework to organize multiple issues that impacted the VIGILANCE intervention (summarized in Figure 4) [18,19]. A detailed analysis of the constructs of the CA framework is included in Multimedia Appendix 1.

### People

This includes issues encountered by the key stakeholders: nurses and patients.

#### Nursing Workflow

Process changes related to the VIGILANCE study protocols resulted in changes to nursing workflows on the study wards. Upon receiving a newly transferred study subject, the nurse assigned to the study patient had to: (1) determine whether the patient was randomized to the standard care or the intervention arm; (2) connect the patient to the monitor; (3) carry the pager; (4) respond to any alarm notifications; and (5) enter the alarm information on the study form. Nursing staff compliance with the study was assessed using the alarm event forms. Among the scanned alarm event forms from the intervention arm, 2.33% (24/1030) were blank without any entry by the nurses and 22.91% (236/1030) of the forms could not be found, indicating decreased compliance with the research practice standards despite multiple in-service sessions. Troubleshooting issues took their time away from actual clinical work.

#### Patient Withdrawal

After starting on the monitoring, 10.68% (110/1030) of patients withdrew from the CPOX monitoring. Of those 110 individuals, 74 did not provide any reason for withdrawal and the remaining 36 patients provided a total of 44 different reasons. The reasons in the comment section captured various other causes for patient withdrawal. These reasons were categorized and presented in (Table 1).

**Table 1:** Reasons for patient withdrawal from continuous monitoring (N=110).

Reason	Number of patients, n (%)^a^
No reason provided for withdrawal from monitoring	74 (67.27)
Probe cable	10 (9.07)
Too many false alarms	6 (5.4)
Uncomfortable probe	6 (5.4)
Restriction in ambulation	4 (3.6)
Noise or beeps	4 (3.6)
Confusion or anxiety	3 (2.7)
Monitor malfunction	3 (2.7)
Sleep disturbance	2 (2.7)
Allergic to Velcro	1 (0.9)
Carpal tunnel	1 (0.9)

^a^Percentage calculated will add up to more than 100% as patients reported more than one reason for withdrawal.

### Organization Issues

This includes challenges associated with the wireless network connectivity and the monitoring technology.

#### Wireless Network Connectivity

Research personnel and HITS staff reported that the fundamental structure of the wireless network had the greatest negative impact on VIGILANCE study implementation. The central station failed to display the data being recorded by the oximeters because of a failure in connection at some point in the long cascade of communication (Figure 1). The hospital had upgraded to a newer wireless structure just before the use of these monitors, and a firmware was installed by Covidien to connect to this newer wireless network. This was thought to have caused the connectivity issue initially. Once the monitor lost wireless connectivity, the network connection required reauthentication for security purposes, but the firmware did not optimally support this function. In the hospitals that did not require reauthentication, the firmware was not required for the monitors to connect to the network. This prevented the bedside monitor from connecting promptly to the central monitor and led to nurses taking more time in some cases and in other cases failing to connect the monitor. Although the access points were installed to create a WLAN, the monitors in the rooms farther from the central station had more difficulty in connecting to the WLAN. The CPOX monitors in both E4 and F4 would alternate between the wireless channels 1, 6, or 11 by default and were later set permanently to only channel 6, which resulted in somewhat better stability. Along with unsupported wireless adapter firmware inside the monitor, other medical devices that were connected to the WLAN increased the traffic and caused interference. Interference from nonmedical devices, such as microwaves and other wireless devices, was thought to be another cause for the wireless CPOX failing to connect to the WLAN [24].

#### Monitoring Technology Issues

Out of 1030 patients, 369 reported at least one monitoring-related issue, and a total of 380 issues were identified. The list of monitoring technology issues for which quantitative data were available is presented below (Table 2).

If a patient’s SpO_2_ was >90% and they were not bradycardic or tachycardic, the alarm was considered to be false. Among the intervention patients, 30.87% (318/1030) had at least one episode of false alarm. The most common reason for false alarms was movement of the probe. These false alarms resulted in notifications being sent to the nurses, with the nurses going back to the patient room to examine the patient and leading to both a disruption in their workflow and alarm fatigue. Failure of the bedside CPOX monitor to connect or respond was considered a monitor malfunction. Malfunction constituted 9.75% (36/369) of the monitoring technology issues and led to 3.49% (36/1030) of patients receiving standard monitoring.

**Table 2:** Reported monitoring technology issues (N=369).

Issue	Number of patients, n (%)^a^
At least one false alarm	318 (86.17)
Monitor malfunction	36 (9.75)
Stopped using monitor due to probe or probe cable	16 (4.33)
Stopped using monitor due to false alarms	6 (1.62)
Stopped using monitor due to constant beeping or noise	4 (1.08)

^a^Percentage calculated will add up to more than 100% as patients reported more than one monitoring technology issue.

Constant beeping or noise from a monitor occurred when it was unable to connect to an access point. This issue led to 1.08% (4/369) of the patients with monitoring technology issues to discontinue use of the monitor. An uncomfortable probe or probe cable resulted in 1.55% (16/1030) of the patients withdrawing from the wireless monitoring system. The research personnel and the nurses reported that the bedside monitor was too large for patient rooms. The dimensions were 8.4 cm × 26.4 cm × 17.3 cm [15]. Although the monitor itself was not that big, the broad base of the monitor stand, the intravenous stand, and a chair in the cramped patient cubicle made the nurses feel as if the monitors were bulky.

### Implementation Issues

The study design planning and signing off on the ethics approval application of the study did not require the nursing managers to assess the change in workflow prior to study commencement. The ward nurses and HITS team were not part of the study design or planning, and feedback from the nurses was only sought after the study was completed. This led to difficulty managing changes in the nursing workflow and delays in detecting connectivity issues.

### Standards and Funding Issues

The frequency that is internationally followed for the Industrial, Scientific, and Medical (ISM) band is between 2.4 and 2.5 GHz, which is not exclusive for medical devices and thus leads to the issue of interference [10]. Congestion caused by multiple medical and nonmedical devices (eg, microwaves and nonhospital devices such as mobile phones) trying to connect to the WLAN resulted in connectivity issues [24]. Research study requirements changed the workflow for the nurses and added extra requirements to their regular practice standards, and there was a lack of funding for involving front-line nurses as part of the study team to lead the project on the wards.

### Quality, Use and Net Impact

An evaluation of impact of the meso and macro level issues on the constructs of the micro level is included in Multimedia Appendix 1 and summarized in Figure 4. The key meso and macro level issues identified during the VIGILANCE study impacted system, information, and service quality at the micro level that led to: (1) decreased use; (2) suboptimal system access; (3) decreased care coordination; and (4) decreased effectiveness and efficiency of the system. The wireless connectivity issues and monitor malfunction affected access through a decreased ability of the nurses to make complete use of the monitors, patient withdrawal, change in nursing workflow, false alarms, wireless connectivity, and probe issues affected the care quality of the trial intervention through decreased effectiveness, and productivity was affected by interference with care coordination. Thus, the decreased quality of the eHealth solution led to decreased clinical adoption by stakeholders.

## Discussion

### Overview

This evaluation report examining the key challenges impacting the implementation of the VIGILANCE trial identified multiple issues in people, organization, implementation, standards, and funding dimensions of the CA framework [19]. Key issues included nursing workflow changes, patient withdrawal, wireless network connectivity, false alarms, monitor malfunction, probe issues, and wireless network standards. These issues led to decreased net benefits and thus decreased clinical adoption of the monitoring system.

### Comparison with Prior Work

Ross et al’s [2] systematic review discussed factors that influenced eHealth systems in clinical environments. Factors such as the ability of eHealth interventions to adapt to the local environment, system functionality, implementation climate, stakeholder engagement, and stakeholder knowledge and beliefs are consistent with the issues that were identified in this study [2].

In the article by Soomro and Cavalcanti [16], they studied the challenges and opportunities associated with the use of WLAN in hospital environments. The 802.11a and 802.11g wireless network standards, which operate on the distributed coordinated function, work on the random-access mechanism where multiple analog and digital signals are combined and transmitted randomly [16]. When there is an overlap of these signals, the channels will randomly retry to transmit after some time, which might lead to the loss of real-time data [16]. To address this lack in Quality of Service (QoS) support, some of the proposed solutions include: (1) extensions such as 802.11e that can provide priority QoS-based access depending on the type of signal (voice, video, best-effort, background traffic) and parameter-based QoS (allots channel time to each station); (2) guaranteed QoS for distinctive traffics; (3) differentiated services architecture based on traffic and QoS guarantees; and (4) integrated networks with both WLAN and wireless personal area networks [16,25]. Some additional factors that affected WLAN connectivity include: coexistent interference from other devices operating in the same ISM band, different configuration requirements for various devices, and the increasing use of mobile devices [16,26]. With the increasing use of mobile and wireless technology in health care, hospitals must update their infrastructure accordingly [26]. Wireless medical device manufacturers must ensure that devices can coexist with other devices prior to their approval for premarket submission, according to the current guidelines by Food and Drug Administration in the United States [27]. Standards for coexistence and the testing of coexistence of wireless medical devices are currently being developed [27,28]. International groups and the Continua Health Alliance have been formed and are collaborating to standardize medical devices and transmission of data [10,29].

Literature has shown that having a comprehensive approach that involves the stakeholders during the planning of any eHealth implementation yields better results, with increased buy-in, improved workflow, and acceptance of the system [2,30]. In eHealth projects, issues with change management and omission to test the system prior to implementing have led to project failures [31,32]. User testing before implementation ensures that the system works according to plan and facilitates user buy-in with the digital intervention [31,33]. Champions of the systems have also been identified in the literature as crucial components of eHealth intervention implementation [2,30]. Therefore, involving users in planning the workflow and testing and engaging front-line nurses who could act as champions of the wireless system monitoring would have facilitated the VIGILANCE team in identifying any system issues, streamlining the workflow, and engaging the nurses more efficiently.

False alarms and constant beeping led to patients withdrawing from the continuous monitoring system and interfered with the nursing workflow. Alarm fatigue is a major concern in the hospital environment with the increasing use of monitoring technology in the hospitals, as desensitization of the health care providers due to constant exposure to alarms, beeps, and other noises can put the patient’s safety at risk [4,34]. False alarms from the CPOX due to motion have been a significant concern over the years [35]. A cableless oximetry probe is a potential solution to remove hindrances to patient ambulation after surgery [33]. With the recent improvements in motion-resistant technology and algorithms, manufacturers are now using new techniques to address this issue [36].

Lau et al used the CA framework to evaluate the impact of electronic medical records postimplementation in an ambulatory care clinic [19,21]. Various evaluation studies, including systematic reviews, have used this framework to understand technology adoption in different clinical settings [19,37,38]. The CA framework offered a multilevel, interrelated view of the various issues impacting the VIGILANCE intervention implementation.

With future trends towards improvements in biosensors, wireless technology, Bluetooth and radio-frequency identification, more wireless devices capturing multiple physiological parameters are being developed and marketed [4,10,39]. Soon, these monitors will make it possible to monitor all the vital signs on regular hospital wards that are currently routinely monitored in the ICU. It will be important to evaluate this technology carefully to ensure it functions in a way that clinicians expect and in a reliable manner [31,32].

### Limitations

A key limitation of this report is that the need to evaluate the impact of factors that might have affected the VIGILANCE study was conceived post study design, and thus we do not have event numbers for all the issues. As this report looked at issues impacting just a single intervention, they might not be generalizable to other eHealth interventions. Future evaluations could include formal evaluation throughout different phases of the project to enhance eHealth intervention implementation and stakeholder management.

### Lessons Learned

The findings from this study support the significance of giving importance to not only health outcomes but also to evaluating the process and people aspects of eHealth research projects to overcome challenges and to optimize the use of eHealth intervention. The results from this study have key implications in a clinical setting. The assessment of challenges shows that it is essential for the originators of eHealth research projects to ensure that the stakeholders, such as nurses, other health care providers, and information and technology staff, are consulted in planning and implementing the intervention, establishing the workflow, and testing the intervention in the already existing hospital infrastructure. Identifying champions among the involved stakeholders and having them as leaders of a research project is crucial for better stakeholder engagement and successful eHealth project completion. Medical device manufacturers are encouraged to consider alarm fatigue while providing configuration and display features for their devices. The lessons learned from this study can help future eHealth research implementation projects. Key issues and potential solutions are summarized in (Table 3).

**Table 3:** Key issues and potential solutions for eHealth research projects.

Issues	Potential solutions
Issues with stakeholder engagement and change management	Involve key stakeholders in planning, establishing workflows, and user testing. Project originators should identify champions and involve them to lead the projects from front-line.
Monitoring technology issues	Usability testing in the actual hospital environment prior to project implementation.
Wireless connectivity	Test for interference and connectivity in the actual environment prior to procuring wireless medical devices.
False alarms	Medical device manufacturers are encouraged to consider alarm fatigue while providing configuration and display features.

### Conclusion

Lau et al’s CA framework was a useful tool for categorizing and understanding the impact of the issues that influenced the deployment of the intervention in the VIGILANCE study. The wireless network in the hospital was demonstrated to be a critical enabler for eHealth interventions. Devices should be chosen based on the available bandwidth and the ability of the device to coexist with other connected devices, and alarm fatigue should be considered while configuring medical devices. Managing change, establishing workflows, testing usability, and engaging stakeholders are key factors in deploying new digital health solutions aimed at improving the process of care and ultimately patient outcomes. The complexities surrounding the implementation of digital interventions should be taken into consideration along with the clinical outcomes while planning eHealth research studies.

## Appendix

Multimedia Appendix 1 Evaluation of impact of issues on the measures of the clinical adoption framework [19].

## Abbreviations

CA: clinical adoption
CPOX: continuous pulse oximetry
eHealth: electronic health
GHz: gigahertz
HITS: health information technology services
ICU: intensive care unit
IEEE: Institute of Electrical and Electronics Engineers
ISM: industrial, scientific, and medical
Mbps: megabits per second
QoS: quality of service
PR: pulse rate
REDCap: Research Electronic Data Capture
SpO_2_: blood oxygen saturation
VIGILANCE: VItal siGns monItoring with continuous puLse oximetry And wireless cliNiCian notification aftEr surgery
WLAN: wireless local area network
