# Supplementary material for: Challenges With Continuous Pulse Oximetry Monitoring and Wireless Clinician Notification Systems After Surgery: Reactive Analysis of a Randomized Controlled Trial
Source: JMIR Med Inform. 2019 Oct 28;7(4):e14603. doi: 10.2196/14603 (PMC6913744; doi:10.2196/14603)
Supplement: Multimedia Appendix 1 [file medinform_v7i4e14603_app0.pdf]

SUPPLEMENTAL MATERIAL

Multimedia Appendix: Evaluation of impact of issues on the measures of the clinical adoption framework [19].

Levels	Dimension	Category	Evaluation of measures
**Meso**	People	Stakeholders, their expectations, roles and responsibilities	Nursing issues – the research study requirements, connectivity issues and false alarms led to changes in the nurses' workflow.<br>Patient issues – false alarms, probe cable, uncomfortable probe and other issues led to patients withdrawing from continuous monitoring.
	Organization	Infrastructure, technology structure	Wireless network connectivity – interference with other medical and non-medical devices led to connectivity issues.<br>Monitoring technology issues – impacted micro level factors negatively.
	Implementation	Project	Daily visit by the research nurse to the study wards to troubleshoot and support the nurses improved the nursing engagement as the study progressed. But front-line nurses and hospital information and technology services staff were not involved in planning and as part of the study leadership team.
**Macro**	Standards	Technology standards, Practice standards	Wireless medical devices interact at the same ISM band as that of microwaves and other non-hospital wireless devices such as mobiles; this increases traffic and causes interference.<br>Research study requirements changed the workflow for the nurses and added extra requirements to their regular practice standards.
	Funding	Remunerations	There was a lack of funding to involve front-line nurses as part of the study team.
**Micro**	System Quality	Functionality – Ease of use, features	Probe cable prevented the device from being completely wireless; this also interfered with patient ambulation.<br>Wireless connectivity issues made it difficult for nurses to connect the devices.<br>The monitor size and its bulkiness made it

			inconvenient for the patients and nurses in the already cramped patient's cubicles.
		Performance – Accessibility, reliability, response time	The constant beeping and false alarms affected the nurses from trusting the system and its reliability.<br>The prolonged time the monitors took to connect to the wireless network and failing to connect impacted its response time.
	Information Quality	Content – Accuracy, completeness, comprehension	The false alarms affected the accuracy of the alerts the nurses were receiving at the central station.<br>The missing alarm event forms indicate that the information was not completely used, and the instructions were not followed. The blank alarm event forms might suggest that the nurses might not have comprehended the information correctly.
		Availability – Timeliness, consistency	Although the alarms were received promptly by the nurses, the constant beeping and false alarms made it inconsistent. This led to nurses ignoring the beeping and contributed toward alarm fatigue.
	Service Quality	Responsiveness – Ongoing support, training	The research team and the HITS team provided ongoing support and tried to resolve the issues. The Covidien technical support team also provided support to address the issues. But, even with updates and installation of custom firmware, the monitors ran into wireless connectivity issues.
	Use	Use behavior pattern – Type, location and flexibility of actual usage	The monitors were stored in the storage area on the same floor as the study ward and were easily available for the nurses. The central monitor was conveniently located in the nursing station.<br>As connecting the monitors to the patients and establishing the wireless connection took a long time, there was disruption to the nurses' workflow leading to decreased use of these monitors.

		Intention to use – Reasons for non-users who became users	With the research team doing in-services and stressing on the importance of patient safety, more nurses started using the system. The nursing staff who saw the real potential of the use of the monitoring system in detecting respiratory depression also became users. But the various issues with the system changed some users to non-users.
	Care Quality	Patient safety – Preventable adverse events	The purpose of the CPOX was to record and notify the nurses if the patient had abnormal readings. With various issues interfering, the intervention patients did not receive complete monitoring that they were assigned.<br>The research data entry took time away from patient care for the nurses.
		Appropriateness and effectiveness – Adherence and compliance with practice standards	The missing and blank alarm event forms indicates that not everyone was compliant with the research practice standards.
	Access	Ability to access service – Availability, timeliness	The wireless connectivity issues prevented the monitors from being available all the time and decreased its timeliness.
		User participation – Patient and nurse participation	The missing and blank alarm event forms indicate that the monitoring system was not used optimally. Among the patients who received the intervention, 10.4% of them withdrew from continuous monitoring after starting it due to various reasons.
	Productivity	Efficiency – Provider resource use, improved patient management	With the nursing staff running into issues with the monitoring system regularly, there was no optimum use of the monitoring system, and hence all the intervention patients did not receive the monitoring entirely as planned. This led to the decreased efficiency of the system.
		Care coordination – Care provision, continuity of care across continuum	The issues prevented the nurses from providing continuous care.
